# Emerging Trends in Pediatric Disease Management and Preventive Care: A Systematic Review

**DOI:** 10.7759/cureus.117058

**Published:** 2026-09-28

**Authors:** Aravindan Kalaivendan, Thiyagaraajan Visveswaran, Suvadha Krishnan, Lasya Marupudi, Ashish Kumar Shukla, Jacqueline A Shah

**Affiliations:** 1 Department of Pediatrics, Kauvery Hospital, Chennai, IND; 2 Department of Pediatrics, Aarupadai Veedu Medical College and Hospital, Vinayaka Mission's Research Foundation (Deemed to be University), Puducherry, IND; 3 Department of Pharmacy Practice, Sri Venkateshwara College of Pharmacy, affiliated with Osmania University, Hyderabad, IND; 4 Department of Radiodiagnosis, Santosh Medical College, Santosh Deemed to be University, Ghaziabad, IND; 5 Department of Pediatrics, Indira Gandhi Institute of Child Health, Bengaluru, IND

**Keywords:** artificial intelligence, digital health, pediatric care, preventive medicine, systematic review

## Abstract

Child health systems are undergoing rapid transformation driven by biomedical innovation, digital integration, and strengthened preventive strategies. Over the past decade, advances in vaccination, pharmacological therapies, digital monitoring, artificial intelligence-assisted diagnostics, and school-based health programs have reshaped pediatric care delivery. Despite expanding technological capacity and structured prevention models, evidence remains fragmented across clinical, behavioural, and diagnostic domains. Long-term effectiveness, sustainability, and equitable implementation of emerging tools remain insufficiently synthesised within a unified framework. This systematic review synthesises contemporary evidence published between 2015 and 2025 to examine evolving approaches to pediatric disease management and prevention. A structured protocol guided database searches, study selection, and quality appraisal. Randomised controlled trials, cluster trials, feasibility studies, and diagnostic accuracy studies involving pediatric populations were included. Risk of bias was assessed using validated appraisal frameworks appropriate to the study design. Ten studies met the eligibility criteria. Interventions demonstrated measurable improvements in vaccine efficacy, respiratory infection reduction, obesity-related outcomes, injury prevention, asthma adherence, and diagnostic accuracy using artificial intelligence models. Digital and behavioural strategies showed promising trends, although long-term validation and scalability data were limited. These findings underscore the convergence of prevention-focused frameworks and technology-supported care models. Strengthening implementation strategies, harmonising outcome reporting, and prioritising equitable access will be essential for optimising future pediatric health systems.

## Introduction and background

The management of pediatric diseases and preventive care has evolved significantly over the last decade because of scientific innovation, changing disease patterns, and rapid technological advances [1]. Pediatric healthcare now addresses the continuing burden of infectious diseases alongside increasing chronic and lifestyle-related conditions, requiring greater integration of disease management and prevention [2-4]. Vaccination remains a cornerstone of pediatric preventive care and has contributed substantially to reductions in morbidity and mortality from vaccine-preventable diseases [5]. Advances in immunology, molecular platforms, and vaccine delivery have further strengthened responses to infectious threats in children [6-8]. Within this review, emerging trends refer specifically to contemporary clinical, preventive, behavioural, digital, and diagnostic approaches intended to improve disease prevention, early detection, treatment effectiveness, and health outcomes in pediatric populations.

The changing burden of pediatric disease extends beyond infectious conditions. Childhood overweight and obesity are increasingly important because of their long-term cardiometabolic and psychosocial consequences [9,10]. School-based, community-based, and family-focused preventive strategies, including physical activity and nutritional interventions, have therefore become important components of pediatric health promotion [11,12]. Chronic respiratory diseases, particularly asthma, also remain important contributors to pediatric healthcare utilisation [13]. Technology-supported approaches, such as electronic inhaler monitoring, have expanded opportunities to improve treatment adherence and disease self-management [14]. Broader developments in nutrition education, health-promotion strategies, and school-based physical activity interventions provide additional context for preventive pediatric care [15,16], although these were not treated as independent evidence domains in the present review. AI and machine-learning applications are increasingly being investigated for pediatric screening and diagnosis [17]. Imaging-based algorithms have demonstrated promising diagnostic performance in areas represented in the contemporary literature, including pediatric respiratory imaging and ophthalmological screening [18]. Such approaches may facilitate earlier recognition of disease, particularly where specialist expertise is limited [19]. Their clinical implementation, however, requires attention to algorithm transparency, representative datasets, external validation, and appropriate regulatory oversight [20].

Preventive pediatric care also increasingly emphasises early identification of risk and intervention during critical developmental periods [21,22]. Socioeconomic and geographic inequalities can influence access to healthcare and pediatric outcomes, making equitable implementation an important consideration [23]. Collectively, advances in vaccination, obesity prevention, chronic disease monitoring, behavioural interventions, and AI-assisted diagnostics indicate a transition toward more proactive and prevention-oriented pediatric care. However, evidence across these areas remains fragmented because interventions differ substantially in their populations, designs, outcomes, and intended purposes. Important uncertainties also remain regarding long-term effectiveness, safety, feasibility, scalability, and external validity. A focused synthesis of recent interventional and diagnostic evidence is therefore needed to clarify the effectiveness and limitations of these emerging approaches within a common pediatric framework.

Objective of the review

This systematic review aimed to synthesise recent evidence on clinical, preventive, behavioural, and diagnostic interventions in pediatric populations from infancy through adolescence. Comparators included placebo, usual care, control interventions, or reference standards, where applicable, and outcomes of interest included clinical effectiveness, safety, adherence, feasibility, behavioural change, and diagnostic performance.

## Review

Methodology

Study Design

This systematic review was conducted and reported in accordance with the Preferred Reporting Items for Systematic Reviews and Meta-Analyses (PRISMA) 2020 guidelines [24]. Eligible designs included randomized controlled trials, cluster-randomized trials, feasibility cluster-randomized trials, and diagnostic accuracy/model-validation studies involving pediatric populations. Feasibility studies were included when they prospectively assessed implementation and reported measurable feasibility and/or clinical outcomes, as they provided early evidence on emerging interventions. Different study designs were interpreted separately according to their research questions and outcome types. Clinical treatments, preventive strategies, behavioural programmes, and technology-based diagnostic solutions were evaluated. Observational studies without an interventional or diagnostic assessment, reviews, editorials, and studies with inadequate outcome information were excluded. Study selection, data extraction, risk-of-bias assessment, and qualitative synthesis were conducted using a predetermined methodology. Quantitative synthesis was considered, but no sufficiently comparable subset of studies was available in terms of interventions, populations, outcomes, and follow-up periods; therefore, narrative synthesis was used. The review protocol was not prospectively registered in International Prospective Register of Systematic Reviews (PROSPERO) or another public repository.

Search Strategy

The literature search was conducted in PubMed/MEDLINE, Scopus, Web of Science, and Google Scholar. Search terms combined pediatric population terms with intervention and technology concepts. The core Boolean search strategy was: (“pediatric” OR “paediatric” OR “child” OR “children” OR “adolescent”) AND (“vaccination” OR “obesity” OR “injury prevention” OR “asthma” OR “artificial intelligence” OR “machine learning” OR “screening” OR “digital health” OR “physical activity”). This strategy was adapted to the indexing conventions and syntax of PubMed/MEDLINE, Scopus, Web of Science, and Google Scholar, with controlled vocabulary used where applicable. Eligible studies were restricted to peer-reviewed English-language publications from January 1, 2015, to December 31, 2025, to capture contemporary developments in pediatric clinical, preventive, and technology-supported care. The applied eligibility filters included publication period, English language, pediatric population, and eligible study designs according to the predefined inclusion criteria. The English-language restriction was applied because reliable translation resources were not available; however, this restriction may have excluded relevant evidence published in other languages. Reference lists of potentially eligible studies were also screened for additional studies.

Eligibility Criteria

Eligible studies included pediatric populations from infancy through adolescence and evaluated a clinical intervention, preventive strategy, behavioural programme, or diagnostic approach with measurable health-related outcomes. Eligible study designs were randomized controlled trials, cluster-randomized trials, feasibility cluster-randomized trials, and diagnostic accuracy/model-validation studies. Diagnostic studies were limited to AI- or deep learning-based approaches used for pediatric screening or classification, as represented by chest radiograph classification and retinopathy of prematurity screening in the included evidence. Studies were required to report quantitative outcomes such as efficacy, risk reduction, change in BMI, adherence, feasibility measures, sensitivity, specificity, or classification performance. Adult-only studies, reviews, editorials, observational studies without an interventional or diagnostic assessment, and studies with insufficient methodological or outcome information were excluded.

Selection of Studies and Data Extraction

All identified records were screened in a stepwise manner. After duplicate removal, titles and abstracts were screened against the predefined eligibility criteria, followed by full-text assessment of potentially eligible articles. Studies meeting all inclusion criteria were included in the qualitative synthesis. Data were extracted using a standardized form that captured author, publication year, study design, population characteristics, sample size, intervention or diagnostic approach, duration of follow-up, and relevant quantitative outcomes. Extracted data were verified by cross-checking the recorded information against the corresponding full-text articles before final tabulation, with inconsistencies re-examined against the original source. The verified data were then summarized in structured tables.

Risk of Bias Assessment

The design of each included study was used to determine the appropriate risk-of-bias tool. The updated Cochrane Risk of Bias 2 (RoB 2) tool was used for individually randomized controlled trials [25], assessing the randomization process, deviations from intended interventions, missing outcome data, outcome measurement, and selection of reported results. Cluster-randomized trials were assessed using the RoB 2 extension for cluster-randomized trials, including consideration of the timing of participant identification and recruitment relative to cluster allocation. Judgments were categorized as low risk, some concerns, or high risk of bias. Risk-of-bias assessments were reviewed against the reported study methods and outcome data, and any uncertainty in domain-level judgments was re-evaluated using the original article before the final assessment was recorded.

Quality Assessment of Diagnostic Accuracy Studies 2 (QUADAS-2) was applied only to the included AI studies that evaluated diagnostic or screening performance, including pediatric chest radiograph classification and retinopathy of prematurity screening. Its use was therefore restricted to studies with a diagnostic accuracy design. QUADAS-2 domains included patient selection, index test, reference standard, and flow and timing, together with applicability concerns [26]. Risk-of-bias findings were considered when interpreting the reliability and generalisability of the evidence.

Data Synthesis

The approach to summarisation of findings across the included studies involved narrative synthesis because the interventions, populations, and outcome measures were too heterogeneous. The quantitative data were obtained and presented in organised tables to enable the effect sizes, efficacy percentages, and diagnostic performance measures to be compared. To better interpret the results, clinical interventions, technology-enabled interventions, and behavioural interventions were analysed under different thematic domains. Meta-analysis was not carried out because of the methodological and clinical heterogeneity between the studies. The synthesis was directed at identifying patterns in effectiveness, magnitude of benefit, and consistency of outcomes, while taking into consideration the study design and risk-of-bias assessment. A formal certainty-of-evidence assessment was not performed because the included studies differed substantially in design, interventions, outcomes, and evidence domains, limiting the applicability of a single certainty framework. Interpretation therefore considered study design, risk of bias, consistency of findings, and applicability of the evidence.

Results

Search Results

The database searches identified 252 records. After removal of 41 duplicates, 211 records underwent title and abstract screening, of which 164 were excluded. Forty-seven full-text articles were assessed for eligibility, and 37 were excluded, leaving 10 studies for qualitative synthesis. Full texts were evaluated using the predefined eligibility criteria for pediatric clinical interventions, preventive strategies, and technology-assisted diagnostic or monitoring approaches. No additional eligible studies were identified through reference-list screening. Figure 1 presents the PRISMA 2020 study-selection process, including identification, screening, eligibility assessment, and final inclusion.

**Figure 1:**
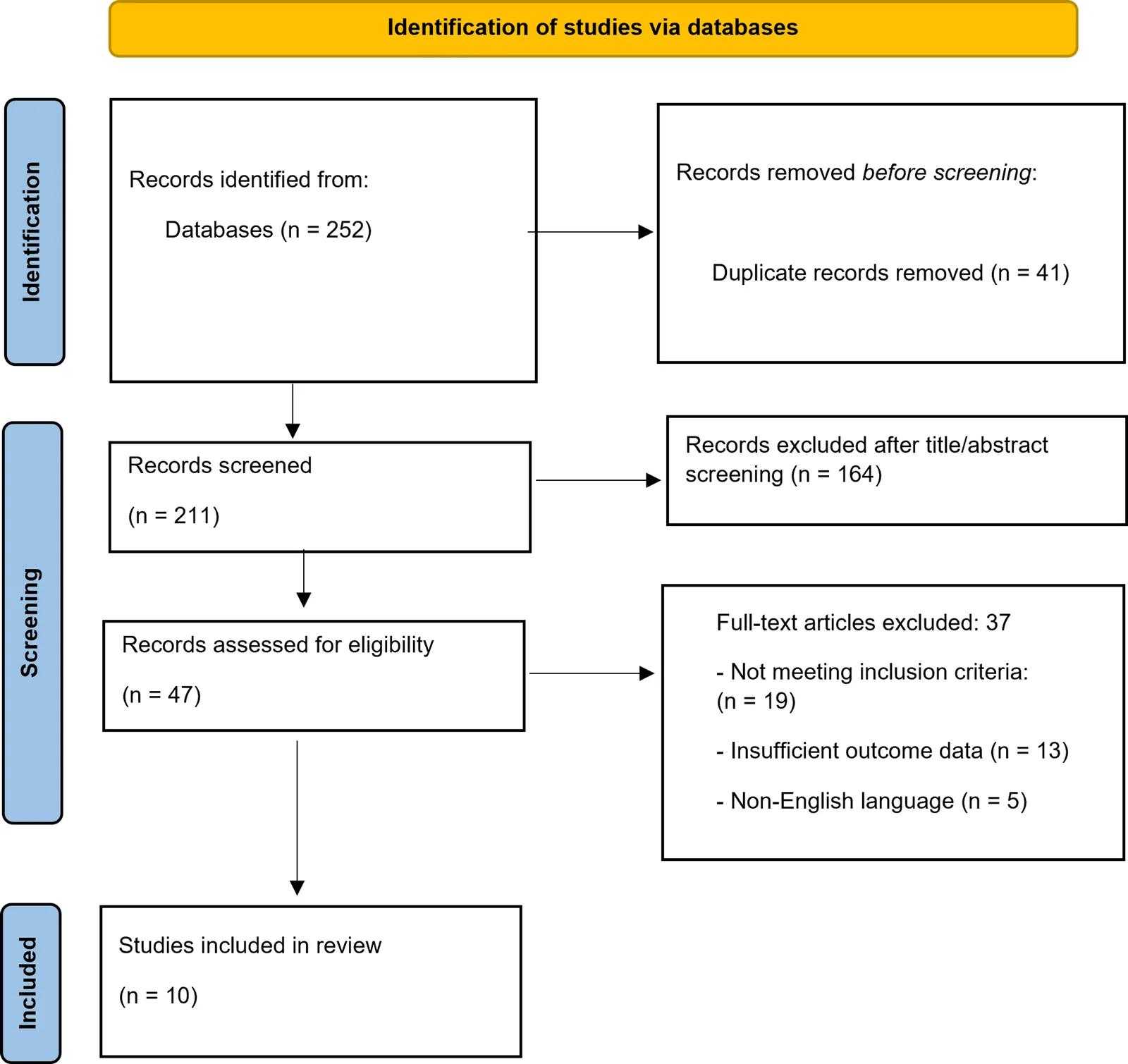
PRISMA flow diagram of the study selection process. PRISMA: Preferred Reporting Items for Systematic Reviews and Meta-Analyses.

Study Characteristics

The 10 included studies were randomised controlled trials, cluster-randomised trials, feasibility trials, and diagnostic accuracy studies involving pediatric populations ranging from infancy to adolescence. The interventions included vaccines, monoclonal antibody prophylaxis, pharmacological obesity treatment, injury prevention counselling, school-based obesity policies, radiographic classification using AI, automated retinopathy screening, and promotion of physical activity in preschool settings. Sample sizes varied considerably, ranging from small feasibility studies to large multicentre studies with thousands of participants. The outcomes included safety, efficacy, change in BMI, incidence of injuries, medication adherence, and diagnostic accuracy. Detailed study characteristics are presented in Table 1.

**Table 1:** Main characteristics of the included studies. IAAH: Increasing Active and Healthy; mRNA: Messenger ribonucleic acid; PA: Physical activity; ROP: Retinopathy of prematurity; RSV: Respiratory syncytial virus; TIPP: The Injury Prevention Program.

Study	Study Design	Setting	Population	Sample Size	Intervention	Duration	Primary Outcome(s)	Main Finding
Walter EB et al. (2022) [27]	Randomised, placebo-controlled trial	Multicentre clinical trial	Children 5-11 years	2,268	BNT162b2 COVID-19 vaccine (BioNTech Manufacturing GmbH, Mainz, Germany; Pfizer Inc., New York, NY, USA)	~2.3 months median follow-up	Safety, immunogenicity, and COVID-19 incidence	Safe, immunogenic, and demonstrated high efficacy against COVID-19
Creech CB et al. (2022) [28]	Randomised, placebo-controlled trial	Multicentre clinical trial	Children 6-11 years	4,016	mRNA-1273 COVID-19 vaccine (ModernaTX, Inc., Cambridge, MA, USA)	~2-3 months follow-up	Safety, immunogenicity, and COVID-19 incidence	Robust immune response with an acceptable safety profile
Hammitt LL et al. (2022) [29]	Randomised, placebo-controlled trial	Multicentre clinical trial	Healthy late-preterm and term infants	1,490	Nirsevimab for RSV prevention (AstraZeneca AB, Södertälje, Sweden)	150 days	Medically attended RSV-associated lower respiratory tract infection	Significantly reduced medically attended RSV-associated lower respiratory tract infection
Weghuber D et al. (2022) [30]	Randomised, double-blind, placebo-controlled trial	Multicentre clinical trial	Adolescents with obesity (12-<18 years)	201	Once-weekly semaglutide (Novo Nordisk A/S, Bagsværd, Denmark) plus lifestyle intervention	68 weeks	Change in BMI	Significant BMI reduction compared with placebo
Gupta RS et al. (2021) [31]	Randomised controlled trial	Outpatient pediatric setting	Children with asthma	252	Sensor-based electronic inhaler monitoring system (Propeller Health, Madison, WI, USA)	12 months	Asthma control, healthcare use, and medication adherence	Improved asthma control; higher healthcare use was observed in the intervention group
Chen Y et al. (2021) [32]	Diagnostic accuracy study	Hospital radiology dataset	Pediatric chest radiographs	>5,000 images	Deep learning model for pneumonia classification	Cross-sectional analysis	Classification accuracy (WHO standard)	High diagnostic performance for pediatric pneumonia detection
Wang J et al. (2018) [33]	Diagnostic accuracy study	Retinal imaging dataset	Preterm infants at risk for ROP	>5,000 retinal images	Deep neural network for ROP screening	Cross-sectional analysis	Sensitivity and specificity for ROP detection	High accuracy for automated ROP screening
Ickovics JR et al. (2019) [34]	Cluster randomised controlled trial	12 K-8 public schools	Students (mean age 10.9 years)	595 (analytic sample)	Implementation of school wellness policies (nutrition and/or physical activity)	4 years	BMI percentile trajectory, behavioural outcomes	Influenced BMI trajectory and health behaviours
Perrin EM et al. (2024) [35]	Cluster randomised controlled trial	Four academic medical centres	Parent-infant dyads	781	The Injury Prevention Program (TIPP)	24 months follow-up	Parent-reported injuries	Significant reduction in parent-reported injuries
Al-Walah MA et al. (2024) [36]	Feasibility cluster randomised controlled trial	Preschools	Children aged 3-5 years	29	IAAH preschool-based physical activity intervention	10 weeks	Feasibility, physical activity, and BMI	High implementation fidelity; PA and BMI trends improved but not statistically significant

Risk of Bias Assessment

The risk of bias was determined based on the study design. Randomised and cluster-randomised trials generally had a low risk of bias in the randomisation process and allocation concealment, with some concerns regarding participant and personnel blinding, particularly in behavioural and policy-based interventions. Attrition bias was low in large vaccine and pharmacological trials but was of greater concern in feasibility and school-based investigations. Diagnostic accuracy studies generally demonstrated a low risk of bias in outcome assessment, although there were some concerns regarding external validation and dataset representativeness. Overall, most studies were judged to have a low risk of bias or some concerns. Accordingly, conclusions regarding vaccine efficacy, Respiratory Syncytial Virus (RSV) prevention, and semaglutide-associated BMI reduction were supported by studies at low risk of bias, whereas findings related to asthma monitoring, behavioural and policy interventions, feasibility outcomes, and AI-based diagnostic performance were interpreted more cautiously because of methodological concerns, including participant selection, missing data, dataset representativeness, and limited external validation. The detailed assessment is summarised in Table 2.

**Table 2:** Risk of bias assessment of the included studies. RoB 2: Risk of Bias 2; QUADAS-2: Quality Assessment of Diagnostic Accuracy Studies 2.

Study	Study design	Appraisal tool	Randomization process or patient selection	Deviations from intended interventions or reference standard	Missing outcome data or flow and timing	Outcome measurement or applicability concerns	Selection of reported result	Overall risk of bias
Walter EB et al. (2022) [27]	Randomized, observer-blinded, placebo-controlled trial	RoB 2	Low risk	Low risk	Low risk	Low risk	Low risk	Low risk
Creech CB et al. (2022) [28]	Randomized, observer-blinded, placebo-controlled trial	RoB 2	Low risk	Low risk	Low risk	Low risk	Low risk	Low risk
Hammitt LL et al. (2022) [29]	Randomized, double-blind, placebo-controlled trial	RoB 2	Low risk	Low risk	Low risk	Low risk	Low risk	Low risk
Weghuber D et al. (2022) [30]	Randomized, double-blind, placebo-controlled trial	RoB 2	Low risk	Low risk	Low risk	Low risk	Low risk	Low risk
Gupta RS et al. (2021) [31]	Randomized controlled trial	RoB 2	Some concerns	Some concerns	Some concerns	Some concerns	Low risk	Some concerns
Chen Y et al. (2021) [32]	Diagnostic model development and validation study	QUADAS-2	Some concerns regarding patient selection	Low risk for reference standard	Some concerns	Some concerns regarding dataset representativeness	Not applicable	Some concerns
Wang J et al. (2018) [33]	Diagnostic model development and validation study	QUADAS-2	Some concerns regarding patient selection	Low risk for reference standard	Some concerns	Some concerns regarding external validation and generalizability	Not applicable	Some concerns
Ickovics JR et al. (2019) [34]	Cluster-randomized controlled trial	RoB 2	Low risk	Some concerns	Some concerns	Some concerns	Low risk	Some concerns
Perrin EM et al. (2024) [35]	Cluster-randomized controlled trial	RoB 2	Low risk	Some concerns	Low risk	Some concerns	Low risk	Some concerns
Al-Walah MA et al. (2024) [36]	Feasibility cluster-randomized controlled trial	RoB 2	Some concerns	Some concerns	Some concerns	Some concerns	Low risk	Some concerns

Preventive and Therapeutic Clinical Interventions

A few studies evaluated preventive and therapeutic interventions for infectious diseases, obesity, injury prevention, and chronic respiratory conditions. The pediatric vaccine trials demonstrated favourable safety, immunogenicity, and protective efficacy. Nirsevimab reduced medically attended RSV-associated lower respiratory tract infection in infants, and semaglutide produced a marked reduction in BMI among adolescents with obesity. The Injury Prevention Program reduced parent-reported early childhood injuries, and sensor-based inhaler monitoring improved medication adherence and asthma-related outcomes. Behavioural and preventive interventions showed variable benefits across different settings. Because the included studies differed substantially in interventions, populations, outcome measures, and effect metrics, the findings were synthesised narratively rather than quantitatively pooled.

Technology-Enabled Screening and Behavioural Health Promotion

Digital health strategies and AI-based diagnostic tools were represented among the included studies. Deep learning models demonstrated high diagnostic performance in the classification of pediatric chest radiographs and in automated screening for retinopathy of prematurity. Behavioural determinants of health were addressed through school-based implementation of wellness policies and preschool physical activity interventions. Although feasibility trials demonstrated positive trends in physical activity and BMI, statistical significance was not always achieved. These studies highlight the growing importance of digital technologies, systematic policy interventions, and early-life interventions as part of preventive health systems for children. Because diagnostic and behavioural studies reported different outcome measures, their findings were interpreted separately and were not presented as directly comparable percentage values.

Discussion

Clinical and Therapeutic Interventions

The present systematic review included 10 studies comprising randomized controlled trials, cluster-randomized trials, a feasibility cluster-randomized trial, and diagnostic accuracy/model-validation studies. The vaccine trials demonstrated favourable safety, immunogenicity, and protective effects in children [27,28]. Nirsevimab reduced medically attended RSV-associated lower respiratory tract infection in infants [29], and once-weekly semaglutide combined with lifestyle intervention produced substantial improvement in BMI among adolescents with obesity [30]. Sensor-based electronic inhaler monitoring also improved medication adherence and asthma-related outcomes [31]. The strength of these findings varied according to study design and risk of bias, with the large vaccine, prophylactic, and pharmacologic trials providing comparatively stronger evidence.

Technology-Enabled Diagnosis

AI represented a distinct diagnostic component of the included evidence. Deep-learning approaches demonstrated promising performance for pediatric chest radiograph classification and automated screening for retinopathy of prematurity [32,33]. These studies evaluated diagnostic or screening performance rather than improvements in clinical decision-making or patient outcomes. Their findings should consequently be interpreted as evidence of diagnostic potential rather than direct clinical benefit. Concerns regarding patient selection, dataset representativeness, external validation, and generalizability further indicate the need for validation across diverse pediatric populations before broader clinical implementation.

Preventive and Behavioural Interventions

School-based wellness policies influenced BMI trajectories and health behaviours [34], whereas the Injury Prevention Program reduced parent-reported early childhood injuries [35]. The preschool-based physical activity intervention demonstrated favourable implementation feasibility, although improvements in physical activity and BMI were not statistically significant [36]. These findings suggest that behavioural and population-based interventions may provide benefits, although their effects can vary according to intervention intensity, implementation setting, participant engagement, and duration of follow-up.

Contextual Implications for Pediatric Healthcare

Implementation science, telemedicine, health equity, and broader digital-health developments provide relevant context for interpreting emerging pediatric healthcare approaches but were not independently evaluated as intervention domains among the included studies [37-41]. These areas should therefore be regarded as contextual considerations rather than direct findings of this systematic review. Successful translation of effective interventions into routine practice may depend on healthcare infrastructure, accessibility, workforce capacity, external validation, and population representativeness. Standardised outcome measures and harmonised reporting approaches may further improve comparison and interpretation across future pediatric studies [42].

Overall Interpretation

The included evidence indicates a movement toward preventive, technology-supported, and outcome-oriented pediatric healthcare. Evidence was comparatively stronger for selected biomedical interventions, including vaccination, RSV prophylaxis, and pharmacologic obesity management, whereas behavioural, feasibility, and AI-based approaches require more cautious interpretation. Substantial clinical and methodological heterogeneity means that findings should remain specific to the populations, interventions, and outcomes evaluated rather than being directly compared across all intervention categories. Future studies should prioritise longer follow-up, assessment of long-term safety and effectiveness, standardised outcome reporting, external validation of AI models, cost-effectiveness, and evaluation across diverse pediatric populations and healthcare settings.

Limitations and Future Directions

This systematic review has important limitations. Substantial heterogeneity in study designs, populations, interventions, outcomes, and follow-up periods prevented quantitative pooling and required narrative synthesis. Differences in follow-up duration also limited assessment of long-term effectiveness. Some behavioural, technology-based, and feasibility studies were conducted in limited settings, which may reduce generalizability. The inclusion of both interventional and diagnostic studies further limited direct comparison across outcomes. Additional limitations include variation in healthcare infrastructure, restriction to English-language publications, absence of a formal certainty-of-evidence assessment, and lack of prospective protocol registration in PROSPERO or another public repository.

Future studies should incorporate longer follow-up periods, standardised outcome definitions, and harmonised reporting frameworks to improve comparability across pediatric interventions. Multicenter studies involving diverse populations are required to assess external validity and implementation across different healthcare systems. Further evaluation of cost-effectiveness, equitable access, and independent external validation of AI tools is also warranted. Implementation-focused studies may help determine whether effective interventions can be scaled sustainably without compromising effectiveness, safety, or accessibility.

## Conclusions

This systematic review indicates that biomedical innovation, digital technologies, and organised preventive strategies are increasingly shaping pediatric disease management and preventive care. The included evidence demonstrates a shift toward early intervention, risk reduction, and outcome-oriented care across multiple pediatric settings. Vaccination, obesity management, injury prevention, and structured behavioural interventions showed measurable clinical or preventive benefits. AI-assisted diagnostics and digital monitoring approaches also demonstrated potential to improve disease detection, screening, and chronic disease management. Broader developments, such as telemedicine, provide relevant context for this transition but were not independently evaluated among the included studies. Important uncertainties remain regarding long-term effectiveness, external validation, implementation across diverse healthcare settings, and equitable access. Overall, the evidence supports a transition toward more proactive, prevention-oriented, and technology-supported pediatric healthcare.
